# Bacterial Keystone Taxa Regulate Carbon Metabolism in the Earthworm Gut

**DOI:** 10.1128/spectrum.01081-22

**Published:** 2022-08-16

**Authors:** Guofan Zhu, Olaf Schmidt, Lu Luan, Jingrong Xue, Jianbo Fan, Stefan Geisen, Bo Sun, Yuji Jiang

**Affiliations:** a State Key Laboratory of Soil and Sustainable Agriculture, Institute of Soil Science, Chinese Academy of Sciences, Nanjing, China; b UCD School of Agriculture and Food Science, University College Dublin, Belfield, Ireland; c Laboratory of Nematology, Wageningen University, Wageningen, the Netherlands; University of Massachusetts Amherst

**Keywords:** earthworm gut, keystone taxa, microbial carbon metabolism, bacterial diversity, bacterial community structure, bacterial community

## Abstract

As important ecosystem engineers in soils, earthworms strongly influence carbon cycling through their burrowing and feeding activities. Earthworms do not perform these roles in isolation, because their intestines create a special habitat favorable for complex bacterial communities. However, how the ecological functioning of these earthworm-microbe interactions regulates carbon cycling remains largely unknown. To fill this knowledge gap, we investigated the bacterial community structure and carbon metabolic activities in the intestinal contents of earthworms and compared them to those of the adjacent soils in a long-term fertilization experiment. We discovered that earthworms harbored distinct bacterial communities compared to the surrounding soil under different fertilization conditions. The bacterial diversity was significantly larger in the adjacent soils than that in the earthworm gut. Three statistically identified keystone taxa in the bacterial networks, namely, *Solirubrobacterales*, *Ktedonobacteraceae*, and *Jatrophihabitans*, were shared across the earthworm gut and adjacent soil. Environmental factors (pH and organic matter) and keystone taxa were important determinants of the bacterial community composition in the earthworm gut. Both PICRUSt2 (Phylogenetic Investigation of Communities by Reconstruction of Unobserved States) and FAPROTAX (Functional Annotation of Prokaryotic Taxa) predicted that carbon metabolism was significantly higher in adjacent soil than in the earthworm gut, which was consistent with the average well color development obtained by the Biolog assay. Structural equation modeling combined with correlation analysis suggested that pH, organic matter, and potential keystone taxa exhibited significant relationships with carbon metabolism. This study deepens our understanding of the mechanisms underlying keystone taxa regulating carbon cycling in the earthworm gut.

**IMPORTANCE** The intestinal microbiome of earthworms is a crucial component of the soil microbial community and nutrient cycling processes. If we could elucidate the role of this microbiome in regulating soil carbon metabolism, we would make a crucial contribution to understanding the ecological role of these gut bacterial taxa and to promoting sustainable agricultural development. However, the ecological functioning of these earthworm-microbe interactions in regulating carbon cycling has so far not been fully investigated. In this study, we revealed, first, that the bacterial groups of *Solirubrobacterales*, *Ktedonobacteraceae*, and *Jatrophihabitans* were core keystone taxa across the earthworm gut and adjacent soil and, second, that the environmental factors (pH and organic carbon) and keystone taxa strongly affected the bacterial community composition and exhibited close correlations with microbial carbon metabolism. Our results provide new insights into the community assembly of the earthworm gut microbiome and the ecological importance of potential keystone taxa in regulating carbon cycling dynamics.

## INTRODUCTION

Earthworms are well-known soil macroinvertebrate detritivores that were one of the originally defined ecosystem engineers (1). Their burrowing and feeding habits have great importance for the maintenance of soil structure and fertility, nutrient availability, and soil health in terrestrial ecosystems (2). The majority of the intestinal microbiota in the earthworm gut originates from the soil near the location of earthworm burrowing activity (3). It is commonly believed that there is a close link between the microbiota colonizing the earthworm gut and the indigenous microbiome in soil environments. The immense space and abundant nutrient supply in the intestinal tract of earthworms contribute to the survival of a diverse bacterial community in the gut (4). However, the unique anaerobic condition of the gut creates a suitable microhabitat for anaerobic and facultative anaerobic bacteria and dramatically modifies the diversity and structure of the bacterial community in earthworm intestine (5). For example, the number of anaerobic bacteria in the earthworm gut was reported to be over 10 to 1,000 times higher than that in the soil environment (6). Yet, how management regimens cause fundamental differences in the microbiome profiles between the earthworm gut and surrounding soil has not been fully characterized in realistic field environments (7–9).

The diversity and composition of intestinal microbial communities have been documented to be jointly driven by environmental and biological factors. Several studies have shown that the bacterial community structure in the earthworm gut is mainly influenced by organic carbon and pH (10). The gut bacteria often form highly diverse and complex communities that collectively function as a microbiome (11). Network analysis can infer species correlations in complex bacterial communities and identify the presumed keystone taxa among the myriad of microbial species (12). The core keystone taxa may induce the occurrence and formation of adaptive symbiosis between the host and the gut microbiome and are usually closely correlated with the overall microbial diversity (13). Discovering such keystone taxa is critical to understanding the assembly and stability of microbial communities (14, 15). Although the food source is found to shape the gut microbiome, the associated keystone taxa within the earthworm intestine remain largely unchanged. Therefore, the understanding of keystone taxa will widen our knowledge about the structure of microbiome assemblages in the earthworm gut and the downstream functional consequences for nutrient dynamics.

The earthworm gut is considered to be an important place for nutrient cycling. For instance, concentrations of essential nutrients (carbon, nitrogen, and phosphorus) in the gut are 2 to 5 times larger than those in the adjacent soil environment (16). Intestinal environmental filtering can strongly affect the colonization of bacterial populations and select specific keystone taxa that perform diverse important functions, including nitrogen fixation and denitrification processes (17, 18). The ubiquitous core taxa have ecological importance in structuring community diversity and complex networks and driving belowground nutrient cycling (15, 19). The diversity of specific keystone taxa contributes to enhancing soil organic carbon (SOC) mineralization and accumulation in agricultural ecosystems (20–22). To date, there are few reports on the effects of these keystone taxa on carbon metabolism in the intestinal microenvironment of earthworms.

Here, we aimed to unravel how earthworms shape the bacterial microbiome acquired from soil in their gut in terms of compositional and functional changes under agricultural fertilization regimens. For that purpose, we investigated the bacterial community in the earthworm gut and surrounding soil under manure treatments in an 18-year field experiment. Specifically, the objectives of this study were to (i) describe the diversity and composition of bacterial community in earthworm gut and surrounding soil, (ii) evaluate the driving factors for the gut bacterial community structure, and (iii) elucidate potential mechanisms of keystone taxa regulating carbon metabolism. It was hypothesized that there are significant differences in bacterial community structure between earthworm gut and adjacent soil. We further predicted that the keystone taxa drive the overall changes in bacterial community composition in the earthworm gut and related carbon metabolism.

## RESULTS

### Physicochemical properties of soil samples and earthworm gut contents.

One-way analysis of variance (ANOVA) indicated that fertilization treatment significantly (*P* < 0.05) altered pH, organic matter (OM), total nitrogen (TN), and moisture content (MC) (see Table S1 in the supplemental material). MC was significantly higher under the HMLe condition (earthworm gut under high-manure and -lime treatment) than under the HMe condition (earthworm gut under high-manure treatment), whereas pH and TN were significantly higher under the HMLs condition (adjacent soil under high-manure and -lime treatment) than under the HMs condition (adjacent soil under high-manure treatment). OM contents were significantly (*P* < 0.05) lower in HMs and HMe treatments than HMLs and HMLe treatments. In addition, our results indicated that earthworm gut varied considerably in pH and MC (Table S1). The pH and MC were significantly (*P* < 0.05) increased in earthworm gut (HMe) versus adjacent soil (HMs). However, fertilization treatment showed no significant (*P* > 0.05) impacts on total phosphorus (TP) and total potassium (TK), while earthworm gut had no significant (*P* > 0.05) effects on OM and TN.

### Diversity and composition of bacterial community.

**FIG 1:**
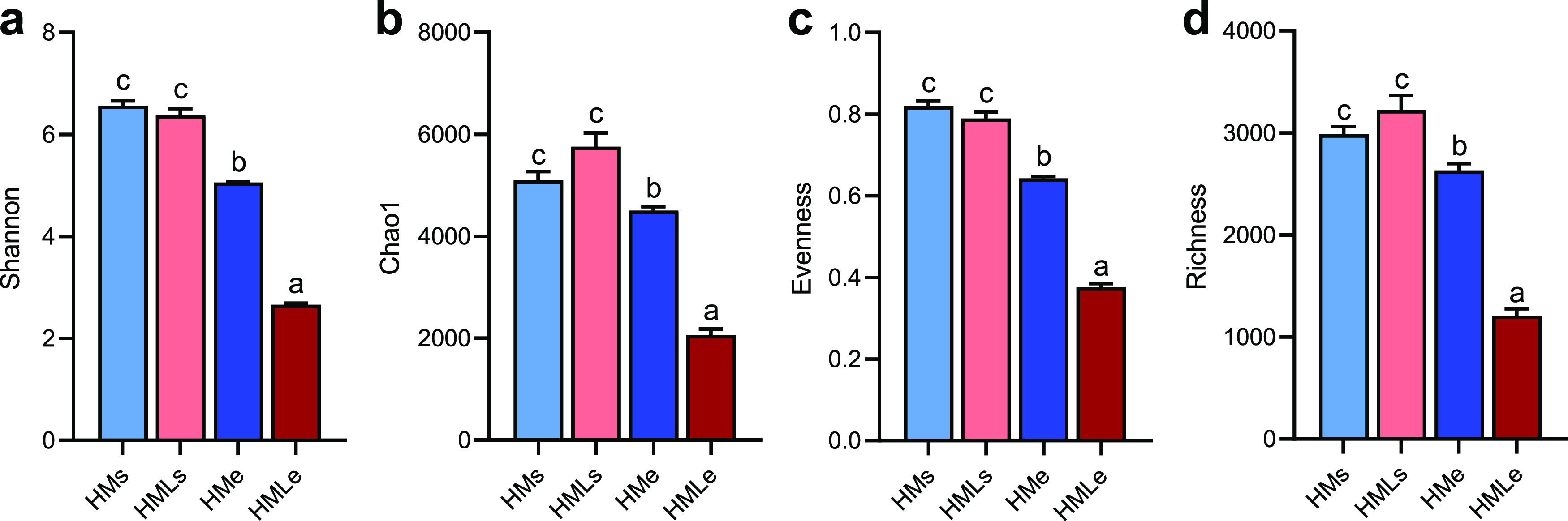
Diversity of the bacterial community in the earthworm gut and adjacent soil. (a) Shannon’s index; (b) Chao1 index; (c) richness; (d) evenness. Different lowercase letters indicate significant differences based on Tukey’s honestly significant difference (HSD) test (*P* < 0.05). HMs, adjacent soil under high-manure treatment; HMe, earthworm gut under high-manure treatment; HMLs, adjacent soil under high-manure and -lime treatment; HMLe, earthworm gut under high-manure and -lime treatment.

Bacterial diversity was significantly changed in the earthworm gut and adjacent soils under different treatments. Overall, the bacterial diversities indicated by the indices of Shannon, Chao1, evenness, and richness were 15.9%, 36.6%, 38.2%, and 40.2% higher in the adjacent soils than those in the earthworm gut (Fig. 1). Furthermore, lime amendment was associated with significantly (*P* < 0.01) reduced intestinal bacterial diversity (52.4% to 63.9%). The bacterial diversity decreased in both earthworm gut and adjacent soil under lime amendment, and the bacterial diversity of earthworm gut changed more than that of adjacent soil.

**FIG 2:**
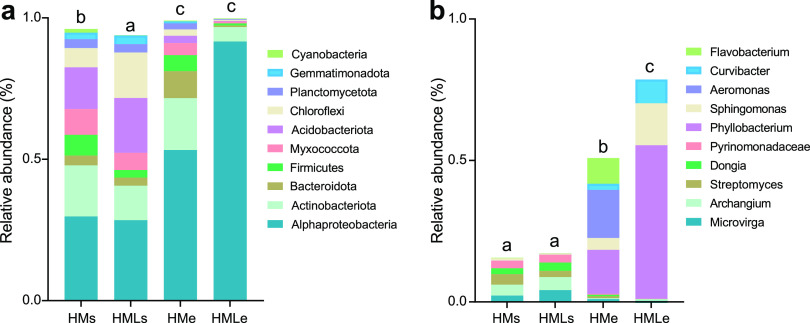
Taxonomic composition of the bacterial community in the earthworm gut and adjacent soil at the phylum (a) and genus (b) levels. Different lowercase letters indicate significant differences based on Tukey’s HSD test (*P* < 0.05). HMs, adjacent soil under high-manure treatment; HMe, earthworm gut under high-manure treatment; HMLs, adjacent soil under high-manure and -lime treatment; HMLe, earthworm gut under high-manure and -lime treatment.

We found that *Alphaproteobacteria* (29.1%), *Acidobacteria* (17.1%), and *Actinobacteria* (15.1%) were the dominant phyla in earthworm gut content, whereas *Alphaproteobacteria* (72.4%), *Actinobacteria* (11.8%), and *Bacteroidota* (5.0%) were dominant in soil (Fig. 2). At the genus level, *Archangium* (4.2%), *Microvirga* (3.2%), and *Streptomyces* (2.9%) were the dominant genera in the adjacent soil, while *Phyllobacterium* (35.0%), *Sphingomonas* (9.5%), and *Aeromonas* (8.5%) were the dominant genera in the earthworm gut (Fig. 2). Canonical correspondence analysis (CCA) indicated that pH, OM, bacterial diversity, and keystone taxa were important determinants of the bacterial community composition (*P* < 0.01) (Fig. S1). The taxonomic composition of the bacterial communities differed significantly between the earthworm gut and adjacent soil (*P* < 0.01) (Fig. 2). Furthermore, lime amendment considerably affected the bacterial community composition in the earthworm gut, as indicated by the significant differences (*P* < 0.05) in the compositions of the bacterial communities under HMe and HMLe treatments.

**FIG 3:**
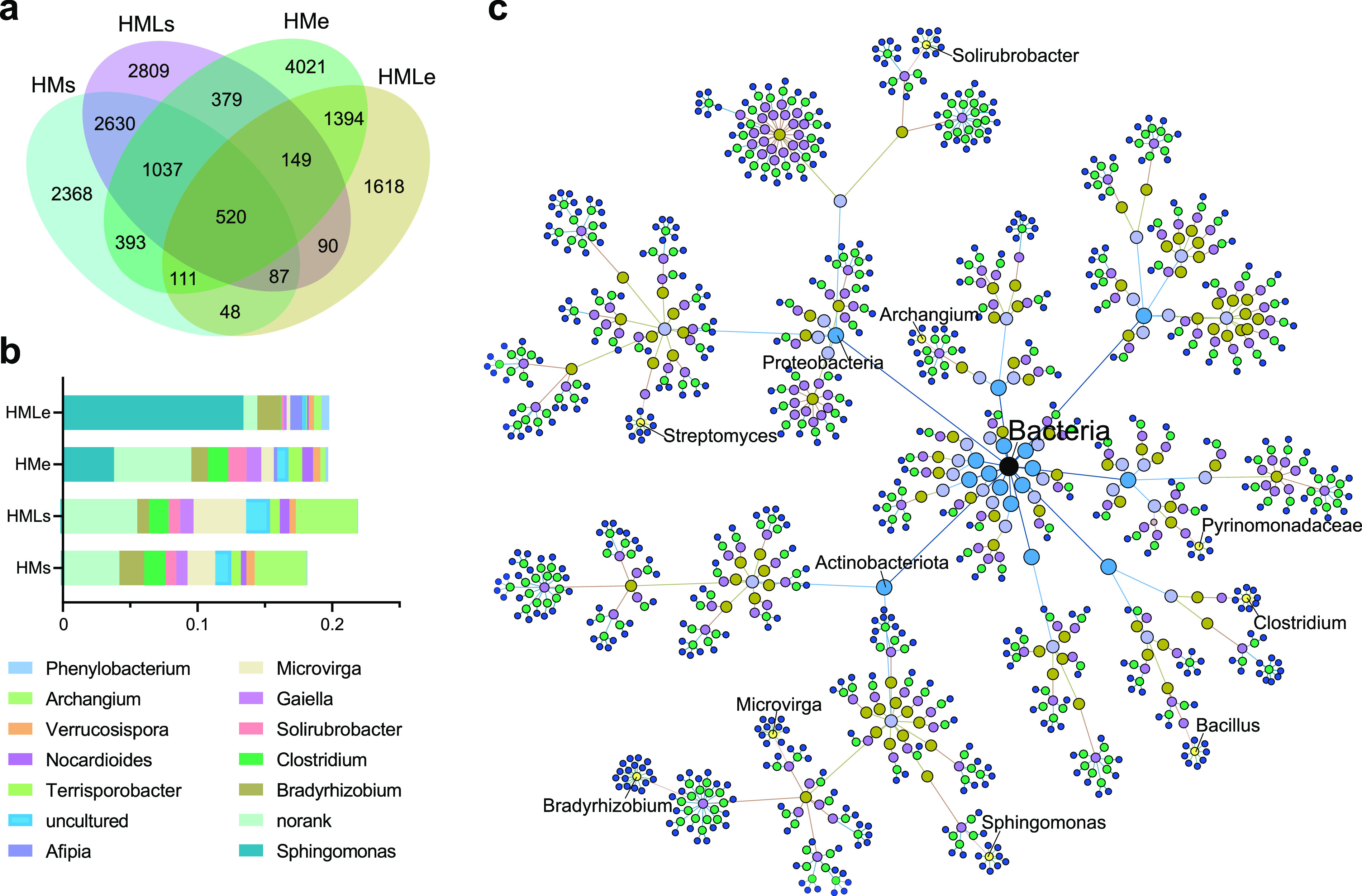
Common and endemic bacterial taxa in the earthworm gut and adjacent soil. (a) Venn diagram showing the number of unique and shared OTUs in the earthworm gut and adjacent soil under manure treatments; (b) composition and abundance of 520 shared OTUs in soil and earthworm gut; (c) taxonomic trees of the shared OTUs. Different colors represent the classification levels of kingdom, phylum, class, order, family, genus, and species. HMs, adjacent soil under high-manure treatment; HMe, earthworm gut under high-manure treatment; HMLs, adjacent soil under high-manure and -lime treatment; HMLe, earthworm gut under high-manure and -lime treatment.

The shared operational taxonomic units (OTUs) between HMs and HMe accounted for 15.7% (2,061/13,137) of total OTUs, mainly belonging to *Clostridium* (2.1%), *Bradyrhizobium* (1.7%), *Streptomyces* (1.6%), and *Pyrinomonadaceae* (1.2%). The shared OTUs between HMLs and HMLe accounted for 7.8% (846/10,827) of total OTUs, primarily affiliating with *Microvirga* (2.2%) and *Bradyrhizobium* (1.3%) (Fig. 3). The 687 OTUs with significant (*P* < 0.01) differences in the earthworm gut and adjacent soil were classified as *Aeromonas* (133 OTUs), *Bradyrhizobium* (74 OTUs), *Clostridium* (38 OTUs), *Phyllobacterium* (33 OTUs), *Streptomyces* (25 OTUs), and *Bacillus* (19 OTUs) (Fig. S2).

### Microbial carbon metabolism.

The metabolic functions of the microbial community were predicted by the abundance of functional pathways using PICRUSt2 and FAPROTAX analyses. According to PICRUSt2 analysis, the distinct carbon metabolism of bacterial communities between earthworm gut and adjacent soil under different treatments included arginine and proline metabolism (1.5%), glycine, serine, and threonine metabolism (1.3%), butanoate metabolism (1.2%), pyruvate metabolism (1.02%), fatty acid metabolism (1.0%), amino acid-related enzymes (1.1%), and valine, leucine, and isoleucine degradation (1.0%) (Fig. S3). According to FAPROTAX analysis, the distinct carbon metabolism between earthworm gut and adjacent soil was mainly related to chemoheterotrophy (34.7%), fermentation (4.5%), hydrocarbon degradation (0.9%), phototrophy (0.6%), chitinolysis (0.6%), and cellulolysis (0.4%) (Fig. S3).

**FIG 4:**
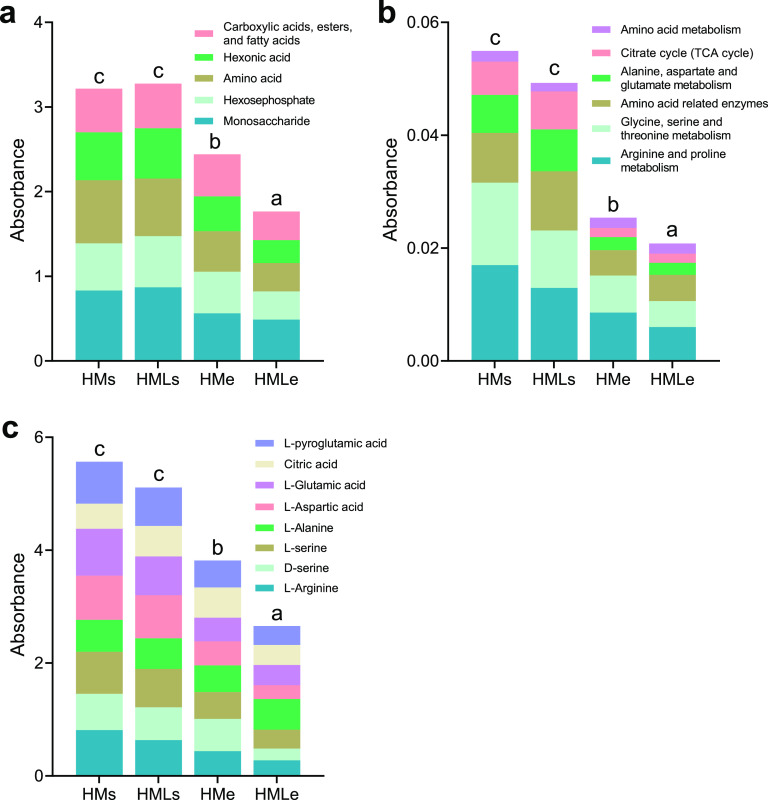
Carbon metabolism of the microbial community. (a) Carbon source metabolic activity indicated by the average well color development (AWCD) in the earthworm gut and adjacent soil under manure treatments; (b) relative abundance of carbon source-related metabolic pathways in the prediction of functional pathways using PICRUSt2 analysis; (c) AWCD values of individual carbon sources determined by Biolog assay. Different lowercase letters indicate significant differences based on Tukey’s HSD test (*P* < 0.05). HMs, adjacent soil under high-manure treatment; HMe, earthworm gut under high-manure treatment; HMLs, adjacent soil under high-manure and -lime treatment; HMLe, earthworm gut under high-manure and -lime treatment.

Based on Biolog analysis, the average well color development (AWCD) was measured to verify microbial carbon metabolism in the earthworm gut and adjacent soil. Results showed significant (*P* < 0.05) differences in amino acids and monosaccharides between adjacent soil and earthworm intestine, as well as carboxylic acids, esters, fatty acids, hexonic acid, and hexosephosphate (Fig. 4a). The average AWCD value was significantly (*P* < 0.01) decreased from 0.51 in HMe to 0.38 in HMLe, but did not vary significantly (*P* > 0.05) between HMs (0.71) and HMLs (0.72). Similar to the predicted pathways of carbon metabolism, the AWCD value was significantly (*P* < 0.05) higher in adjacent soil than in earthworm gut (Fig. 4b and c). Furthermore, the microbial carbon metabolism in both adjacent soil and earthworm gut decreased significantly (*P* < 0.05) with lime application.

### Potential keystone taxa mediated carbon metabolic activity.

**FIG 5:**
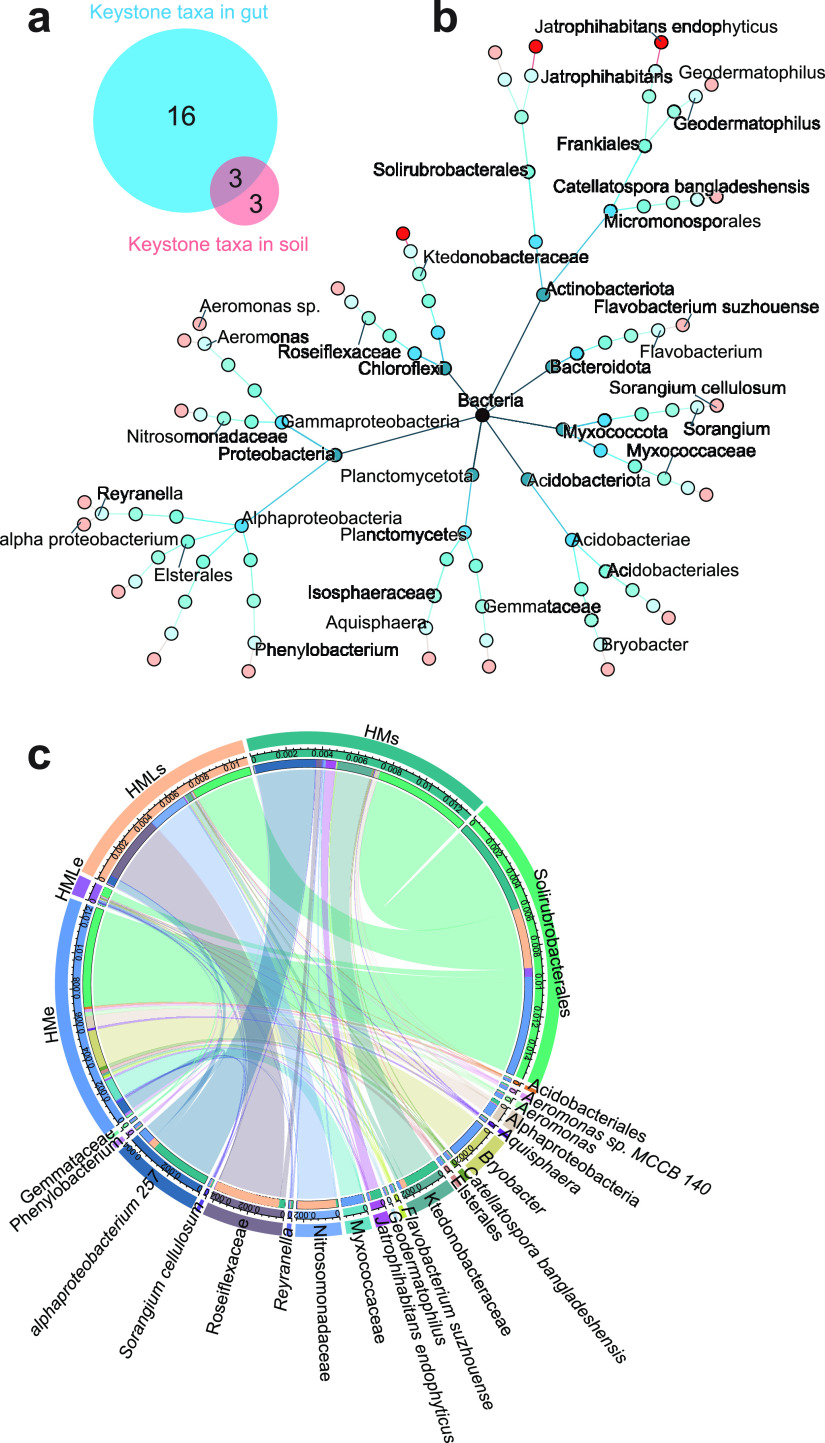
Structural composition of keystone taxa. (a) Venn diagram of unique and shared keystone taxa in the earthworm gut and adjacent soil; (b) systematic classification tree of keystone taxa. Different colors represent the classification levels of kingdom, phylum, class, order, family, genus, and species. Bright red nodes represent the shared keystone taxa in the earthworm gut and adjacent soil. (c) Chord diagram indicating the composition and relative abundance of keystone taxa in the earthworm gut and adjacent soil under manure treatments. HMs, adjacent soil under high-manure treatment; HMe, earthworm gut under high-manure treatment; HMLs, adjacent soil under high-manure and -lime treatment; HMLe, earthworm gut under high-manure and -lime treatment.

The degree of connectivity of individual nodes within (*Z_i_* degree) and among (*P_i_* degree) the modules was calculated to identify the 22 keystone taxa that belonged to *Reyranella* (0.13%), *Bryobacter* (0.06%), *Jatrophihabitans* (0.02%), *Aeromonas* (0.02%), *Catellatospora* (0.01%), and *Phenylobacterium* (0.01%) across treatments (Fig. 5; Fig. S4). Three shared keystone taxa between soil and earthworm gut were *Solirubrobacterales*, *Ktedonobacteraceae*, and *Jatrophihabitans*. The relative abundance of keystone taxa was significantly (*P* < 0.05) higher in soil than that in earthworm gut. Lime application significantly decreased the abundance of bacterial keystone taxa in the earthworm gut by about 20 times, yet it had no significant effect on the abundance of keystone taxa in the adjacent soils (Fig. 5c). The removal of keystone taxa led to a significant (*P* < 0.05) decline of the network stability, as indicated by the modularity values (Fig. S4c). Correlation analysis showed that both pH and OM exhibited significant (*P* < 0.05) correlations with the relative abundance of keystone taxa, which were mostly negative for pH and mostly positive for OM (Fig. S4d).

**FIG 6:**
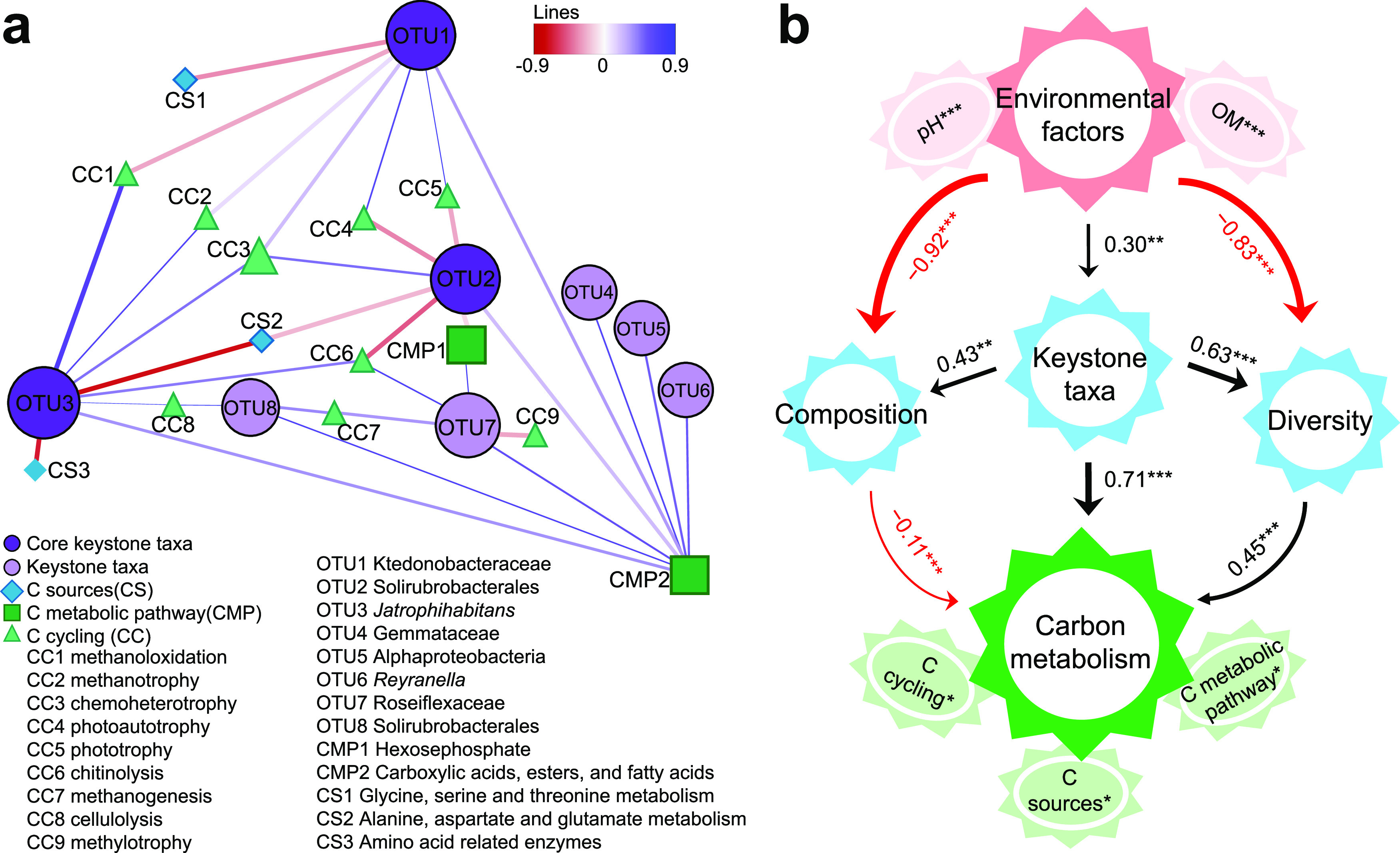
Effects of keystone taxa on microbial carbon metabolism. (a) Correlation analysis between keystone taxa and carbon metabolism indicated by PICRUSt2, FAPROTAX, and Biolog analysis. Carbon (C) cycling is predicted by PICRUSt2 analysis, and the C metabolic pathway is predicted by FAPROTAX analysis. C sources are indicated by the average well color development (AWCD), which was measured by Biolog assay. The shapes of nodes represent different units, and the size indicates the relative abundance of carbon metabolism or degree of connectivity of bacterial OTUs. Red edges represent negative correlations, while blue edges represent positive correlations. The thickness of each connection is proportional to the value of Spearman’s correlation coefficient. (b) Contributions of environmental factors (pH and OM) and the bacterial community to microbial carbon metabolism determined using structural equation modeling. The bacterial community is indicated by diversity (Shannon index), composition (first axis in canonical correspondence analysis [CCA1]), and keystone taxa (the sum of relative abundance). *, *P* < 0.05; **, *P* < 0.01; ***, *P* < 0.001.

Correlation analysis indicated that the potential keystone taxa *Gemmataceae*, *Alphaproteobacteria*, *Reyranella*, and *Roseiflexaceae* had significant (*P* < 0.05) correlations with carbon metabolism, including methanogenesis, cellulolysis, methylotrophy, hexosephosphate (CMP1), and carboxylic acids, esters, and fatty acids (CMP2). In particular, three common keystone taxa, *Solirubrobacterales*, *Ktedonobacteraceae*, and *Jatrophihabitans*, in soil and earthworm gut were significantly (*P* < 0.05) associated with methanoloxidation, methanotrophy, chemoheterotrophy, photoautotrophy, phototrophy, and CMP2 (Fig. 6a). Random forest modeling combined with correlation analysis consistently indicated that environmental factors (pH and OM) and bacterial community (diversity, composition, and keystone taxa) exhibited significant correlations with carbon metabolism (Fig. S5). Structural equation modeling (SEM) further suggested that environmental factors (pH and OM) had significantly positive (*P* < 0.001) effects on keystone taxa in the earthworm gut, but significantly (*P* < 0.01) negative influences on the diversity and composition of bacterial community. Importantly, keystone taxa may contribute significantly to carbon metabolism not only by direct effect (*r *=* *0.93, *P* < 0.001), but also by indirect influence via mediating the diversity (*r *=* *0.45, *P* < 0.001) and composition (*r* = −0.11, *P* < 0.01) of bacterial communities (Fig. 6b).

## DISCUSSION

### The bacterial community varied in earthworm gut and adjacent soil.

The soil environment provides suitable conditions for earthworms to burrow, feed, digest, and excrete. Earthworms feed selectively on materials rich in organic matter and participate in energy transfer and nutrient cycling dynamics in soil (23). Our results showed that the bacterial community structures are significantly different between the earthworm gut and the surrounding soil habitats. The study species, the endogeic *Drawida* species, is considered to live in semipermanent vertical burrows and relies heavily on soil organic matter as the major food source (24). The feeding preference of earthworms (e.g., litter versus mineral soil feeding) and stable pH environment (neutral or slightly acidic condition) determine the ingested microbiota that will undergo gut passage (9). The ingested microorganisms encounter an anoxic gut environment that is rich in water-soluble organic matter derived from the hydrolysis and degradation of the gut mucus (25) and ingested biomass (16). The anoxic conditions and high concentrations of organic substrates in the earthworm gut provide an ideal and stable space for the survival and colonization of anaerobic or facultative anaerobic bacteria. These dominant bacteria in the earthworm gut are usually a subset of denitrifying and fermentative bacteria, including Escherichia (*Gammaproteobacteria*), *Bacteroeciesides* (*Bacteroidetes*), *Prevotella* (*Bacteroidetes*), and *Bacillus* (*Firmicutes*) (23). The earthworm gut has been described as a “mutualistic digestive system,” in which the exoenzymes produced by ingested microbiome improve the capacity of the earthworm to assimilate nutrients during gut passage (5). The abundant ammonium and amino acids in earthworm gut can enhance the anabolism of ingested microbiome (26). Our findings add new evidence suggesting that while the bacterial community compositions in earthworm gut and soil are interrelated, the anoxic gut hosts a community that is different from the ingested bacterial community, possibly functioning as a mutualistic digestion system for anaerobic metabolism.

### Environmental factors and keystone taxa mediated the bacterial community.

We revealed that the changes of soil pH and OM had strong influences on the composition of the bacterial community in the earthworm gut under fertilization treatments. It is widely accepted that pH and organic carbon are important factors affecting the diversity and structure of the bacterial community (27, 28). Although lime application directly raised soil pH, pH in the gut was relatively neutral and less variable than soil pH, indicating that pH homeostasis occurs in the gut (16). The earthworm intestine secretes various enzymes involved in metabolic processes, including catalase, glutathione *S*-transferase, and superoxide dismutase (9, 29). The pH of animal guts has a strong impact on the activities of intestinal enzymes and contributes considerably to the assembly of intestinal microbiota and the physiological metabolic activities of earthworms (5). In turn, the increased activities of intestinal enzymes may stimulate the digestion of complex organic matter during gut passage. Intestinal mucus contains large quantities of water-soluble organic carbon, which is secreted into the foregut and can be readily degraded by the gut microbiome (30). The majority of bacteria of the gut microbiome rely heavily on the decomposition of fresh organic matter for energy availability. Due to selective feeding, the high concentration of soil organic matter in the anoxic gut is considered a survival resource that modifies the diversity and community structure of the gut microbiome (31). Earthworm mucus contains a variety of carbohydrates and acetate (25), which changes the adjacent soil pH and alters the soil bacterial community and the process of carbon cycling dynamics.

We revealed that keystone taxa were strongly associated with the diversity and composition of the bacterial communities across the earthworm gut and adjacent soil. There is sufficient evidence that keystone taxa can drive the alterations in the structure and network stability within the bacterial community (32–34). Various animal model systems, such as mice (35), zebrafish (36, 37), bees (38), fruit flies (39, 40), and nematodes (41), are often used to identify the potential keystone taxa of intestinal microbiome (13, 42). We provide new data here to extend previous work showing that pH and the availability of organic carbon selectively favor the growth and colonization of keystone taxa across the earthworm gut and adjacent soil, as indicated by the observed significant correlations between potential keystone taxa and environmental factors (pH and OM). A growing body of evidence suggests that the structures and activities of bacterial communities can be indirectly affected by pH and OM via regulating keystone taxa (21, 43). The highly connected keystone taxa have strong predictive power for compositional turnover in the bacterial community suffering from environmental disturbance (44). These statistical keystone taxa are highly connected taxa and may exhibit unique and critical roles in organizing the diversity and structure of the bacterial community (19). The recruitment of particular keystone taxa may induce the competitive interactions between microbial taxa, and individually they may sustain the changes in microbiome diversity and composition better than all taxa combined (21, 44). Additionally, the bacterial associations with keystone taxa may contribute to maintaining community stability more pronouncedly than environmental selection (13, 45). Despite the distinct differences in the conditions of the gut and surrounding soil, the “core” bacterial hub taxa remained relatively stable, with large amounts of connected taxa. These core hub taxa are likely to have a high clustering coefficient with wide niches, which strengthens the connection between bacterial taxa in the gut and adjacent soil.

### Keystone taxa mediated microbial carbon metabolism.

We observed that keystone taxa were significantly correlated with microbial carbon metabolism (Fig. 6), indicating that keystone taxa may play important roles in the activities of carbon metabolism in the earthworm gut and adjacent soil. In particular, *Bacteroides*, *Prevotella*, and *Bacillus* were abundant genera in the earthworm intestine and have been reported to possess functional capabilities for degrading complex carbon sources to support their growth and development (46–49). Our results showed that the rare order *Solirubrobacterales* was related to carbon metabolism of hexosephosphate, and the genera *Reyranella*, *Gemmataceae*, and *Roseiflexaceae* exhibited significant correlations with carboxylic acids, esters, and fatty acids. *Solirubrobacterales* has previously been identified to improve soil organic matter and sustain soil fertility in agricultural ecosystems (50). *Reyranella* spp. are regarded as copiotrophic bacteria and play crucial roles in carbon metabolism under sufficient nutrient conditions (51). The bacterial diversity and composition are of ecological significance for fundamental ecosystem processes, such as microbial carbon metabolism and nutrient cycling (52, 53). Keystone taxa may influence microbial functioning by selectively regulating community diversity and composition, regardless of their abundance (19, 54). In the face of changing pH and OM, these potential keystone taxa contribute directly and indirectly to regulating carbon metabolism, the decomposition and sequestration of organic matter, and microbially driven carbon cycling (20, 22). Keystone taxa also profoundly drive the positive relationships between microbiome diversity and ecosystem functioning by mediating species interactions in communities (21, 45). However, comparatively few studies have tested theoretical predictions about the influence of keystone taxa on the composition and functional capacity of bacterial community under changing environmental conditions. Further studies are required to validate the potential regulatory mechanisms of keystone taxa in the context of whole co-occurrence networks.

### Conclusions.

Here, we characterized the bacterial community and carbon metabolic activity in the earthworm gut and in the adjacent soil under manure treatments. We revealed that the earthworm gut significantly decreased bacterial diversity and altered the bacterial community composition compared to that in the adjacent soil. PICRUSt2 and FAPROTAX combined with Biolog analysis consistently indicated that microbial carbon metabolism in adjacent soil was significantly higher than that in the earthworm gut, regardless of fertilization treatments. The bacterial groups *Solirubrobacterales*, *Ktedonobacteraceae*, and *Jatrophihabitans* were identified as the shared keystone taxa in the gut and exhibited strong correlations with carbon metabolism. We conclude that environmental factors (pH and OM) and keystone taxa jointly regulate microbial carbon metabolism across the earthworm gut and adjacent soil. Our results provide new insights into the community assembly of the earthworm gut microbiome and the importance of potential keystone taxa in regulating carbon cycling dynamics.

## MATERIALS AND METHODS

### Long-term field experiment.

The experimental site was located at the Ecological Experimental Station of Red Soil of the Chinese Academy of Sciences in Yingtan, Jiangxi Province (28°15′20′′N, 116°55′30′′E). The site has a subtropical monsoon climate with a mean annual temperature of 17.8°C, a mean annual precipitation of 1,795 mm, and a mean frost-free period of 212 days. The soil developed from a parent material of quaternary red clay (viscous, humid, iron-rich soil), and is characterized by strong acidity, a poor organic matter content, and low nitrogen and phosphorus storage capacity. The long-term experiment contained two treatments with three replicates, including high manure (HM) (pig manure with 600 kg N ha^−1^ year^−1^) and high manure plus lime (HML) [pig manure with 600 kg N ha^−1^ year^−1^ plus Ca(OH)_2_ applied at 3,000 kg ha^−1^ 3 years^−1^]. Crops of monocropped maize (cultivar Suyu no. 24) had been planted annually in April and harvested in July since 2002. Each plot was 2 m long, 2 m wide, and 1.5 m deep. The pig manure on a dry matter basis contained a total carbon (TC) content of 397.5 g kg^−1^ and total nitrogen (TN) content of 34.5 g kg^−1^.

### Collection of soil samples and earthworm gut content.

A total of 20 earthworms were collected alive from each plot by a simple handpicking method, and corresponding adjacent soil (depth of 0 to 30 cm) was collected in late July 2019. We separated the earthworms and adjacent soil samples into two groups (10/20) under HM and HML treatments. There were 24 samples total, including 12 each of soil and earthworm samples, respectively (2 treatments × 3 replicates × 2 groups). In the following text, soil samples under HM and HML treatments were designated HMs and HMLs, while the earthworm intestinal contents were designated HMe and HMLe, respectively. The collected earthworms were washed five times with sterile water and immediately put into an incubator filled with ice to reduce their activity. The COI gene was amplified to identify the earthworm species using the universal primers LCO1490 and HCO2198 (55). The dominant earthworm species was identified as the endogeic *Drawida* species. The earthworms were surface sterilized with 70% ethanol and washed repeatedly with sterile water. After that, earthworms were put into 30% ethanol solution at 0°C for anesthesia until they no longer wriggled and then were cleaned with sterile water to remove the sticky mucus on the surface. Then, the abdominal intestine of earthworms was cut by a sterilized surgical blade, and the intestinal epidermis was fixed outward. The intestinal contents were scraped off with a blade and collected as the earthworm gut samples (yielding between 63 and 117 mg per earthworm).

### Properties of soil samples and earthworm gut content.

The pH of earthworm gut contents or soil samples was measured in a 1:2.5 gut content-water suspension or 1:2.5 soil-water suspension using a glass electrode (56). Organic matter (OM) was determined by heating with potassium dichromate (57). Briefly, a total 0.5-g sample was decanted into a digestion tube, and 1.2 mL digestion reagent was added. The digestion product was then transferred into a 2.5-mL polystyrene cuvette, and the absorbance was measured at 600 nm on a spectrophotometer. Total nitrogen (TN) was determined by the micro-Kjeldahl method (58). For the digestion, a 0.5-g sample was thoroughly mixed with 5 mL sulfuric acid before heating. After the digestion, boric acid solution was added for distillation, containing methyl red-bromocresol green as a titration indicator for the calculation of N contents. Total phosphorus (TP) was determined by the molybdenum blue method in sulfuric acid (59). The reaction mixture contained acidified ascorbic acid solution and a mixed reagent, including ammonium heptamolybdate tetrahydrate solution, potassium antimony tartrate solution, and 50% sulfuric acid. Absorbance was measured by a spectrophotometer at an 880-nm wavelength. Total potassium (TK) was determined by the atomic absorption spectrophotometer (Analyst 800; Perkin Elmer Instruments, Norwalk, CT) (60). Each sample (0.2 g) was first digested with a 5-mL diacid mixture (HNO_3_-HClO_4_ at 5:1 [vol/vol]) in a fume cupboard and heated at 160°C for 5 h. After complete digestion, the K concentration in the digests was determined by the atomic absorption spectrophotometer. Moisture content (MC) was measured by an oven drying method. The physicochemical properties of the earthworm gut and adjacent soils are shown in Table S1 in the supplemental material.

### Illumina sequencing and bioinformatic analysis.

Total DNA was extracted from 0.5 g (dry weight) of soil samples or earthworm gut content using a DNeasy PowerSoil kit (Qiagen) according to the manufacturer’s instructions. The DNA yields from soil and earthworm gut content samples were 12.8 to 15.1 μg 0.1 g^−1 ^dry weight and 10.6 − 13.8 μg 0.1 g^−1 ^dry weight, respectively. The quality of DNA indicated by 260/230-nm ratios extracted from soil samples and earthworm gut content ranged from 1.60 to 1.70. The V4-V5 region of the bacterial 16S rRNA gene was amplified with the universal primers 515F and 806R (61), and specific barcode was added for PCR amplification. After library preparation, 2 × 300-bp paired-end sequencing reactions were performed on an Illumina MiSeq platform.

Merging of paired-end reads, quality filtering, and taxonomic assignments were conducted using the Quantitative Insights into Microbial Ecology (QIIME version 1.9.1) pipeline (62). Briefly, the paired-end reads were assembled and quality filtered using PANDAseq (63). The merged reads were clustered into operational taxonomic units (OTUs) by clustering at a 97% similarity threshold using the UCLUST algorithm. Taxonomic assignments were conducted using the RDP classifier algorithm by comparing the OTU sequences against the Greengenes database (64). We observed totals of 290,423 and 482,726 sequences from soil samples and earthworm gut content, with averages of 24,202 and 40,227 sequences per sample, respectively. A total of 15,441 OTUs were detected for 12 soil samples and 12 earthworm gut samples, and the average sequence length of optimized reads was 376 bp. Alpha diversity and canonical correspondence analysis of the bacterial community were calculated after rarefying all samples to the same sequencing depth. PICRUSt2 and FAPROTAX were applied to predict the functional potential of the bacterial community by 16S rRNA gene sequencing profiles following the developer’s instructions (65, 66). The PICRUSt2 predictions were mapped to the annotated genes catalog of the Kyoto Encyclopedia of Genes and Genomes (KEGG) database. FAPROTAX extrapolated taxonomic microbial community profiles into putative functional profiles based on a database of cultured microorganisms. Raw sequences were uploaded to the National Center for Biotechnology Information under program accession no. PRJNA810764.

### Carbon source metabolism in microbial communities.

The Biolog Gen III microplates used in this experiment included 71 kinds of carbon sources for measurement of microbial carbon metabolism. Briefly, 2 g of fresh soil or earthworm gut contents was added to a sterilized triangular flask containing 20 mL 0.85% NaCl solution at 25°C for 30 min. After that, 1 mL of microbial suspension was diluted in a final concentration of 1:20 with sterilized 0.85% NaCl solution. Then, 150 μL of each sample was dispensed into each well of a Biolog Gen III plate and incubated at 25°C. The absorbance was measured by a microplate reader at an optical density of 590 nm (OD_590_) every 12 h.

### Network analysis and potential keystone taxa.

Network analysis was performed by calculating Spearman’s correlations between taxa with co-occurrence network (CoNet) inference using Cytoscape software (67). The OTUs found in more than two-thirds of the samples were contained in the analysis. Spearman’s correlations were considered significant when the coefficient (*r*) was >0.8 or <−0.8 and the *P* value was <0.01. The *P* values were corrected using the Benjamini-Hochberg procedure to eliminate false positives (68). The Gephi software was used to display the co-occurrence network (69). Nodes indicated bacterial OTUs, and edges indicate significant connections between nodes. The putative keystone taxa were recognized by calculating the within-module connectivity (*Z_i_*) and among-module connectivity (*P_i_*) (70). The *Z* value quantified to what extent a node connected to other nodes in its own module, while the *P* value quantified how well a node connected to different modules. The module hubs (*Z* > 2.5 and *P* < 0.62, key to its own module connection) and connectors (*Z* < 2.5 and *P* > 0.62, key to network connection) were identified as the potential keystone taxa. Network modularity was measured for clustering using the Newman’s equation (71). High modularity values indicated that more correlations existed within modules.

### Statistical analysis.

One-way analysis of variance (ANOVA) and Spearman correlation analysis were performed in SPSS 24.0 (Chicago, IL, USA). The nonparametric Kruskal-Wallis test (72) and Wilcoxon rank sum test (73) were performed to identify potential markers (OTUs and carbon metabolism) by the linear discriminant analysis effect size (LEfSe) method using the R package ggplot2 (74). Canonical correspondence analysis (CCA) was used to determine the weight of environmental variables on the bacterial community using the R package vegan. A chord diagram was constructed using R package Circlize, and a volcano map of differential OTUs was determined with the screening conditions of a false-discovery rate (FDR) of <0.05 and log_2_ fold change of >|2| using the R package ggplot2.

Random forest modeling was conducted to estimate the contribution of environmental factors and the bacterial community to carbon metabolism in the earthworm gut and adjacent soil. The model was constructed using the R package randomForest (75), and significance was determined using the R package A3 (76). Structural equation modeling (SEM) was conducted to evaluate the direct and indirect contributions of abiotic (pH and OM) and biotic (bacterial diversity and keystone taxa) factors to microbial carbon metabolism, using IBM SPSS AMOS version 22.0. We introduced latent variables as composites for the interpretation in the SEM model. The model fitness was assessed based on a nonsignificant χ^2^ test (*P* > 0.05), the goodness-of-fit index, and the root mean square error of approximation (77).

### Data availability.

The high-throughput sequencing data that support the findings of this study are openly available in National Center for Biotechnology Information under accession no. PRJNA810764.
