# Supplementary material for: Bacterial Keystone Taxa Regulate Carbon Metabolism in the Earthworm Gut
Source: Microbiol Spectr. 2022 Aug 16;10(5):e01081-22. doi: 10.1128/spectrum.01081-22 (PMC9603485; doi:10.1128/spectrum.01081-22)
Supplement: Supplemental file 1 — Fig. S1-S5, Table S1. Download spectrum.01081-22-s0001.pdf, PDF file, 0.7 MB [file spectrum.01081-22-s0001.pdf]

Supplementary Materials for

Bacterial keystone taxa regulate carbon metabolism in the earthworm gut

Guofan Zhu, Olaf Schmidt, Lu Luan, Jingrong Xue, Jianbo Fan, Stefan Geisen, Bo Sun, Yuji Jiang*

*Corresponding author:

Yuji Jiang

E-mail address: yjjiang@issas.ac.cn; Tel: 86-25-86881245; Fax: 86-25-86881000

This PDF file includes:

Figures S1 to S5

Table S1

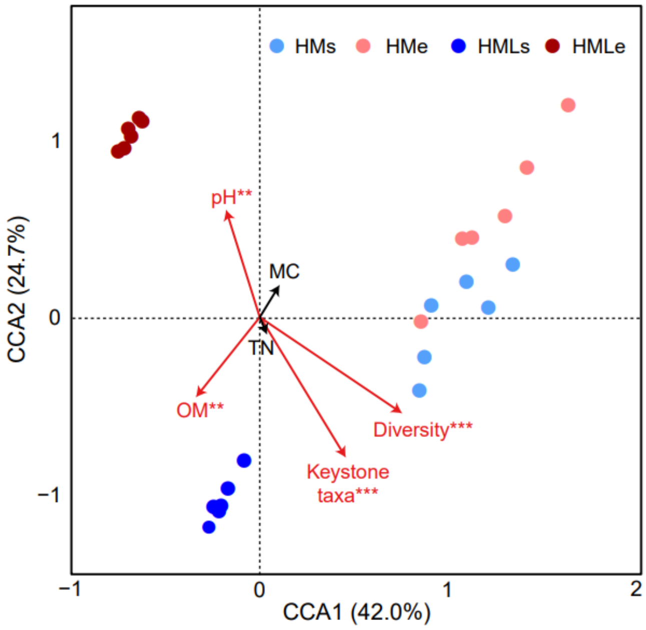

Fig. S1. Canonical correspondence analysis (CCA) indicating the relationships between soil properties and the bacterial communities in the earthworm gut and adjacent soil. The bacterial community is indicated by diversity (Shannon index) and keystone taxa (the sum of relative abundance). MC, moisture content; OM, organic matter; TN, total nitrogen. HMs, adjacent soil under high manure treatment; HMe, earthworm gut under high manure treatment; HMLs, adjacent soil under high manure and lime treatment; HMLe, earthworm gut under high manure and lime treatment. ***, $P < 0.001$; **, $P < 0.01$.

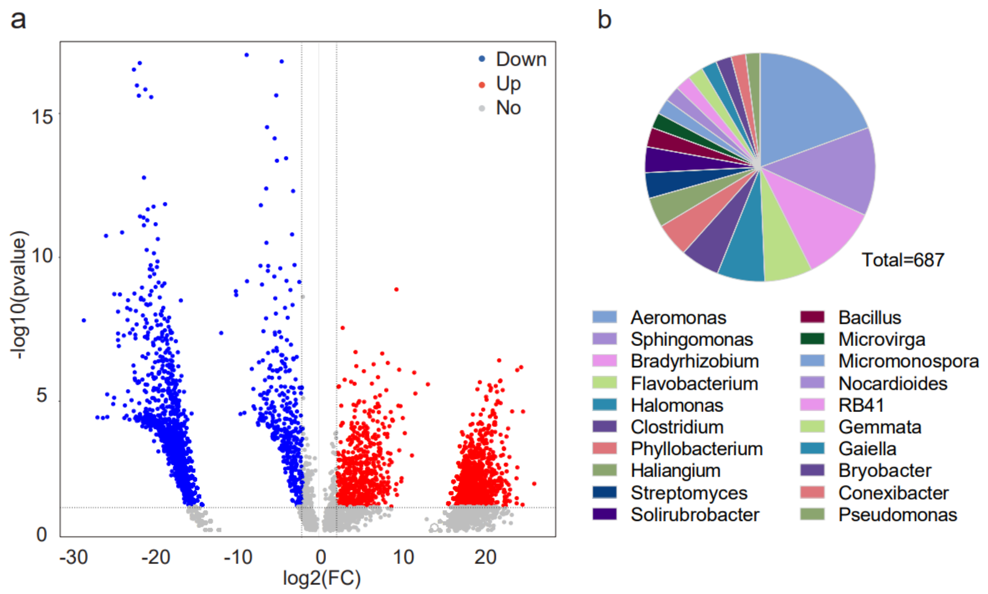

Fig. S2. The differential OTUs in the earthworm gut and adjacent soil. (**a**) Volcano plots showing OTUs significantly ($P < 0.01$) enriched (blue dots) and depleted (red dots) in the earthworm gut compared with adjacent soil as determined by differential abundance analysis. Each dot represents an individual OTU, and the X axis indicates the abundance fold change. (**b**) Pie plot showing the taxonomic composition of 687 differential OTUs at the genus level.

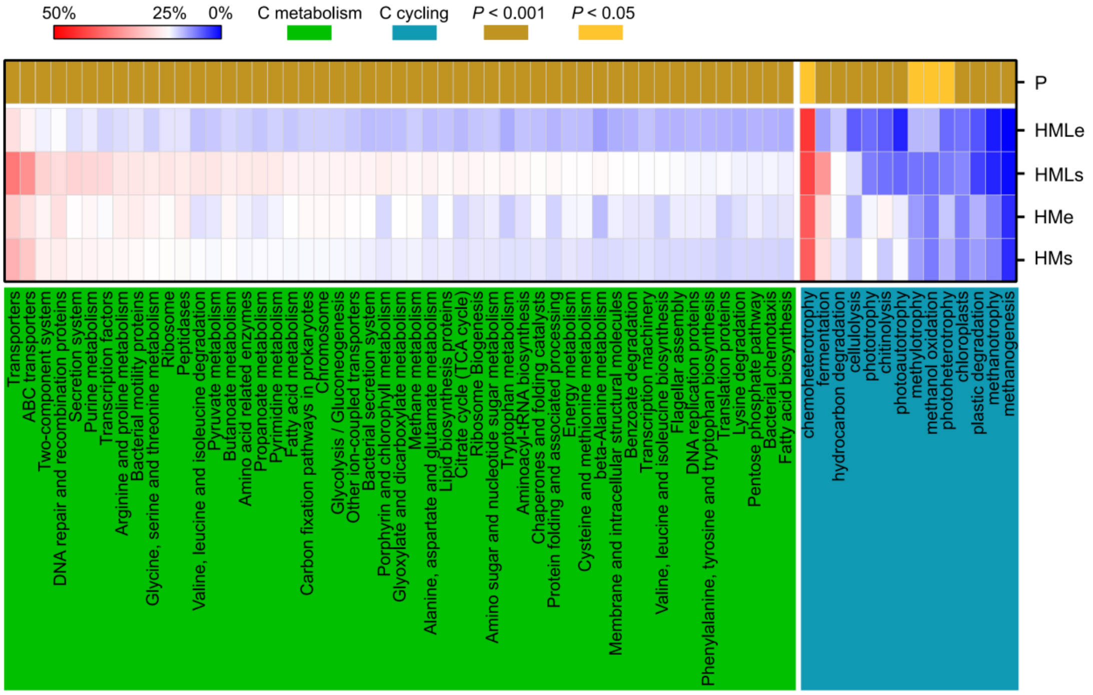

Fig. S3. Heatmap displaying the relative abundance of differential carbon (C) metabolism in the earthworm gut and adjacent soil. The relative abundance of significantly differential carbon pathways. The relative abundance of the differential C cycling was predicted by PICRUSt2 analysis, while that of C metabolic pathways was predicted by FAPROTAX analysis. HMs, adjacent soil under high manure treatment; HMe, earthworm under high manure treatment; HMLs, adjacent soil under high manure and lime treatment; HMLe, earthworm gut bacteria under high manure and lime treatment.

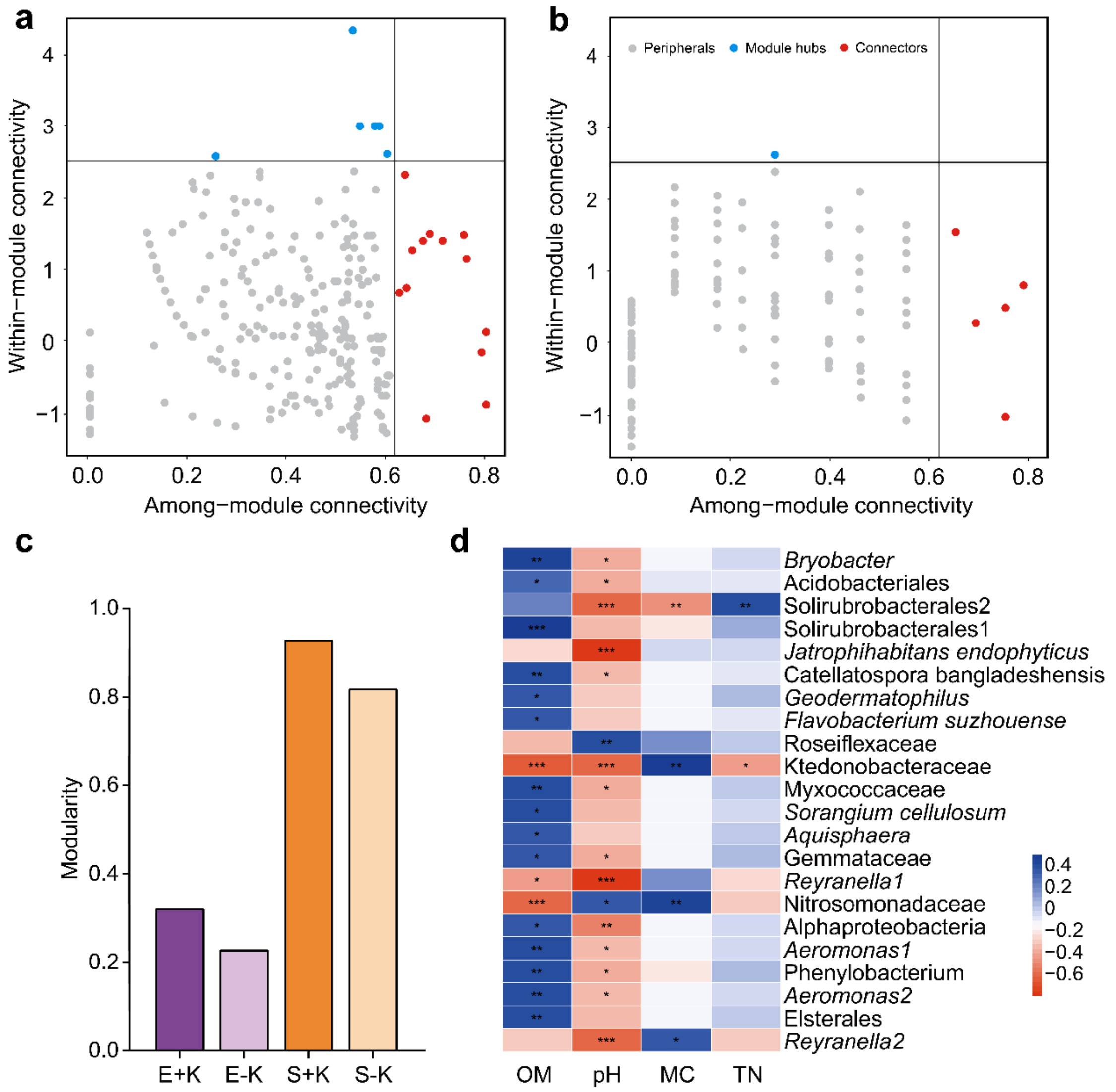

Fig. S4. Keystone taxa and modularity in the co-occurrence networks. *Zi-Pi* plots show the distribution of nodes based on their topological roles in the networks of the adjacent soil (**a**) and earthworm gut (**b**). The module hubs (blue dots, $Z > 2.5$ and $P < 0.62$) and connectors (red dots, $Z < 2.5$ and $P > 0.62$) are categorized as potential keystone taxa. (**c**) The values of modularity are calculated to estimate the influence of keystone taxa on the network stability in the earthworm gut and adjacent soil. E+K and E-K, the network modularity in the earthworm gut before and after removing keystone taxa. S+K and S-K, the network modularity in adjacent soil before and after removing keystone taxa. (d) Correlations between keystone taxa and environmental factors (pH, OM, MC, and TN). OM, organic matter; MC, moisture content; TN, total nitrogen. ***, $P < 0.001$; **, $P < 0.01$; *, $P < 0.05$.

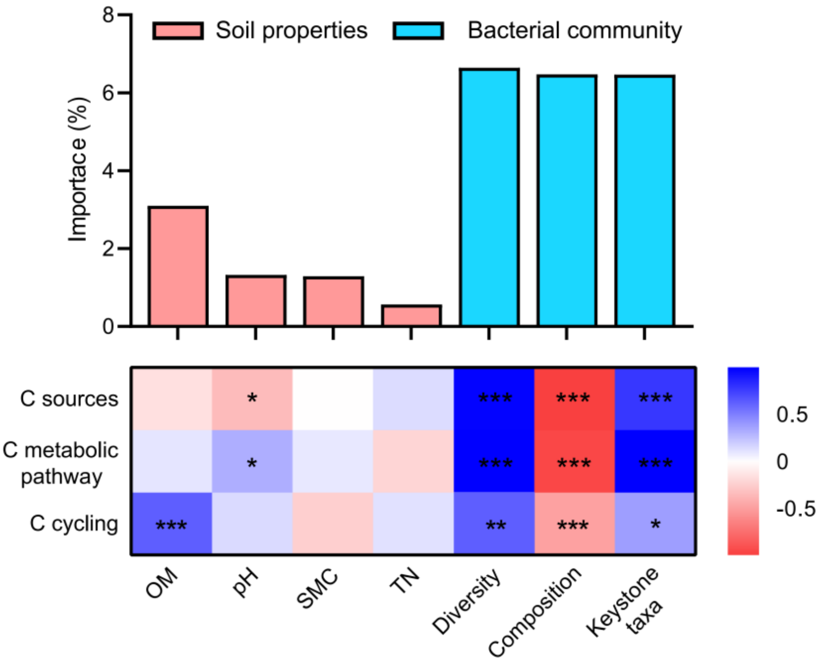

Fig. S5. Contributions of soil properties and the bacterial community to carbon microbial metabolisms based on correlation analysis and random forest modelling. Carbon (C) cycling is predicted by PICRUSt2 analysis, and C metabolic pathway is predicted by FAPROTAX analysis. C sources is indicated by the average well color development (AWCD), which is measured by Biolog analysis. The bacterial community is indicated by diversity (Shannon index), composition (first axis in canonical correspondence analysis, CCA1) and keystone taxa (the sum of relative abundance). Colors represent Spearman's correlation coefficients. MC, moisture content; OM, organic matter; TN, total nitrogen. ***, $P < 0.001$. **, $P < 0.01$. *, $P < 0.05$.

Table S1 The physicochemical properties of soil and earthworm gut content[a].

	pH	OM (g kg^{-1})	TN (g kg^{-1})	MC (%)	TP (g kg^{-1})	TK (g kg^{-1})
HMs	5.69±0.13a	17.45±0.10b	1.12±0.09a	14.43±0.15a	1.72±0.06a	11.69±0.18a
HMLs	7.02±0.17c	14.87±0.08a	1.37±0.04c	14.18±0.31a	1.835±0.04a	11.57±0.05a
HMe	6.31±0.03b	16.77±0.08b	1.19±0.03ab	18.46±0.20b	-	-
HMLe	6.91±0.03bc	14.49±0.14a	1.27±0.05bc	21.48±0.47c	-	-

[a] OM, organic matter; TN, total nitrogen; MC, moisture content; TP, total phosphorus; TK, total potassium. HMs, adjacent soil under high manure treatment; HMe, earthworm gut under high manure treatment; HMLs, adjacent soil under high manure and lime treatment; HMLe, earthworm gut under high manure and lime treatment.
